# PeakPrime: a peak-guided primer design pipeline for target enrichment in 3′-end RNA-seq

**DOI:** 10.1093/bioadv/vbag080

**Published:** 2026-03-19

**Authors:** Franco Poma-Soto, Brecht Soulliaert, Hanne Van Droogenbroeck, Pieter Mestdagh, Jo Vandesompele

**Affiliations:** Department of Biomolecular Medicine, Ghent University, Ghent 9000, Belgium; OncoRNALab, Cancer Research Institute Ghent (CRIG), Ghent University, Ghent 9000, Belgium; Department of Biomolecular Medicine, Ghent University, Ghent 9000, Belgium; OncoRNALab, Cancer Research Institute Ghent (CRIG), Ghent University, Ghent 9000, Belgium; Department of Biomolecular Medicine, Ghent University, Ghent 9000, Belgium; OncoRNALab, Cancer Research Institute Ghent (CRIG), Ghent University, Ghent 9000, Belgium; Department of Biomolecular Medicine, Ghent University, Ghent 9000, Belgium; OncoRNALab, Cancer Research Institute Ghent (CRIG), Ghent University, Ghent 9000, Belgium; Department of Biomolecular Medicine, Ghent University, Ghent 9000, Belgium; OncoRNALab, Cancer Research Institute Ghent (CRIG), Ghent University, Ghent 9000, Belgium

## Abstract

**Motivation:**

Targeted enrichment can offset the bias and depth requirements of random-primed second-strand synthesis in 3'-end RNA-seq by reallocating reads to transcripts of interest. We present PeakPrime, a reproducible Nextflow pipeline that (i) identifies high-coverage 3${\;}^{\prime }$ RNA-seq regions via MACS2, (ii) selects exonic windows around significant peaks, (iii) designs strand-appropriate cDNA primers via Primer3, and (iv) screens specificity with Bowtie2, producing publication-ready plots and quality control (QC) summaries.

**Results:**

We demonstrate coverage-informed primer design using a custom 3${\;}^{\prime }$ protocol that employs a barcoded oligo(dT)-VN primer, optimize parameters on IMR-32 neuroblastoma and Universal Human Reference RNA data, and validate primers by quantitative PCR (qPCR) and targeted 3${\;}^{\prime }$ RNA-seq. Targeted libraries preserve gene-level expression correlations with random-primed 3${\;}^{\prime }$ RNA-seq (Pearson r≈0.88 across conditions), while boosting the fraction of reads assigned to target genes from <1% to ∼25%–42% and increasing detection of the lowest-abundance transcripts. While empirically tested in bulk RNA-seq, PeakPrime enables extensions to single-cell and spatial workflows where oligo(dT) capture is followed by *in situ* reverse transcription and second-strand synthesis.

**Availability and implementation:**

PeakPrime source code, example configs, and documentation are available at https://github.com/OncoRNALab/PeakPrime.

## 1 Introduction

The 3${\;}^{\prime }$-end RNA sequencing (3${\;}^{\prime }$ RNA-seq) is a cost-efficient alternative to whole-transcript RNA-seq for differential gene expression analysis and high-throughput screens: by capturing polyadenylated transcripts with oligo(dT) and sequencing their 3${\;}^{\prime }$ ends, these workflows reduce the required depth per sample while maintaining robust gene-level quantification (Ma *et al.* 2019, Moll *et al.* 2014). Because 3${\;}^{\prime }$ RNA-seq generates only one fragment per transcript, the number of reads mapped to a gene is directly proportional to its expression level, avoiding transcript-length normalization when comparing expression between genes and showing lower impact of RNA integrity. In contrast, conventional RNA-seq typically requires high-quality RNA for mRNA enrichment and can be biased toward longer transcripts that produce more fragments (Moll *et al.* 2014, Alpern *et al.* 2019, Ma *et al.* 2019). Thus, 3${\;}^{\prime }$ RNA-seq provides a reliable and affordable approach to gene expression profiling regardless of transcript length.

Representative implementations include academic and commercial protocols for bulk and single-cell studies. Early-multiplexed approaches enable high-throughput screens with streamlined wet-lab steps. These methods typically perform first-strand cDNA synthesis with a barcoded oligo(dT) primer, followed by pooling, second-strand generation, and library amplification (Moll *et al.* 2014, Lohman *et al.* 2016, Zheng *et al.* 2016, Alpern *et al.* 2019, Pallares *et al.* 2020, Janjic *et al.* 2022).

Often, second-strand synthesis relies on random priming, which introduces non-uniform coverage and sequence-context biases that favor abundant RNAs and penalize low-abundance targets (Fu *et al.* 2014, Hashimshony *et al.* 2016, Bush *et al.* 2017). Classic studies of random hexamer priming revealed strong base-composition artifacts at read starts and uneven transcript coverage; more recent analyses corroborate reverse transcription and priming artifacts as pervasive contributors to quantification bias (Hansen *et al.* 2010, Shi *et al.* 2021).

A practical strategy to mitigate these issues, particularly when rare transcripts matter, is replacing random second-strand priming with target-specific primers that direct amplification toward a defined gene set (Song *et al.* 2021). In adjacent domains, targeted panels are commonplace: single-cell platforms support targeted gene expression panels (Song *et al.* 2021, Chen *et al.* 2022), and *in situ* sequencing based spatial assays identify a large number of RNA-targeting probes by direct imaging of the tissue (Ke *et al.* 2013, Wang *et al.* 2023, Cook *et al.* 2023, Park *et al.* 2023, Kuemmerle *et al.* 2024). Conceptually, introducing gene-specific priming at the second-strand step can reallocate sequencing to transcripts of interest, increasing sensitivity while reducing depth and cost.

Here we present PeakPrime, a Nextflow pipeline that couples statistical peak detection on 3${\;}^{\prime }$ coverage profiles with automated, strand-aware primer design. PeakPrime leverages MACS2 (Zhang *et al.* 2008) to call significant coverage peaks from RNA-seq BAM files, constrains candidate windows to exonic regions around those peaks, designs primers with Primer3 (Untergasser *et al.* 2012), and finally screens specificity using Bowtie2 (Langmead and Salzberg 2012). The pipeline outputs QC tables and publication-ready visualizations, and is portable via Nextflow with pinned software environments.

PeakPrime differs from other primer design tools that either design RT-PCR assays without leveraging 3${\;}^{\prime }$ coverage peaks (Tokheim *et al.* 2014) or use RNA-seq only to prioritize isoforms or genes (Filomena *et al.* 2025). We argue that peak-guided targeting increases on-target fractions, boosts sensitivity for low-abundance genes, and reduces the required sequencing depth relative to random-primed controls in bulk 3${\;}^{\prime }$ data, advantages likely to extend to single-cell and spatial transcriptomics contexts. Here we show that PeakPrime-guided targeted 3${\;}^{\prime }$ RNA-seq preserves gene-level expression and differential expression patterns relative to random-primed controls, while markedly increasing on-target read fractions and detection of low-abundance transcripts.

## 2 Methods

This section describes the experimental and computational procedures used to design, validate, and benchmark primers for target enrichment of low-abundance transcripts in 3${\;}^{\prime }$-end RNA-seq data. The workflow integrates a custom 3${\;}^{\prime }$ library preparation protocol employing a barcoded oligo(dT)-VN primer and short-read sequencing, and the PeakPrime Nextflow pipeline for peak-guided primer design, followed by validation by qPCR and targeted RNA-seq.

### 2.1 PeakPrime: peak-guided primer design

#### 2.1.1 Rationale

Target enrichment in 3${\;}^{\prime }$-end RNA-seq workflows requires primers that bind specifically to the 3${\;}^{\prime }$ regions of transcripts without introducing off-target amplification. Traditional primer design tools either ignore empirical coverage patterns or lack integration with transcript-level specificity screening. PeakPrime addresses this gap by coupling statistical peak detection on RNA-seq coverage profiles with automated, strand-aware primer design and comprehensive quality control. The pipeline prioritizes primers targeting high-confidence 3${\;}^{\prime }$ regions while ensuring compatibility with cDNA amplification chemistry and minimizing cross-reactivity across the transcriptome.

#### 2.1.2 Operating modes

PeakPrime supports three operational modes tailored to different experimental requirements: Peak-based single-peak mode (default) identifies the single highest-confidence coverage peak per gene from RNA-seq data and designs primers targeting that region. This mode is optimal when one primer set per gene suffices and empirical coverage data are available. The pipeline ranks peaks by statistical significance or coverage intensity and selects the best-performing candidate per gene based on configurable quality thresholds.

Peak-based multi-peak mode extracts multiple significant peaks per gene and designs distinct primer sets for each. This mode accommodates genes with complex 3${\;}^{\prime }$ UTR structures, alternative polyadenylation sites, or scenarios requiring broader isoform coverage. An optional distance-weighted optimization algorithm selects optimal primer combinations across peaks by balancing proximity to 3${\;}^{\prime }$ ends, isoform coverage, and peak quality.

Distance-based mode designs primers within a fixed distance from annotated 3${\;}^{\prime }$ transcript ends without requiring RNA-seq input. This mode enables primer design for preliminary experiments, cross-platform applications, or situations where empirical (3${\;}^{\prime }$ end) coverage data are unavailable. Primers are designed on fixed-length templates extracted from transcript sequences.

#### 2.1.3 Pipeline steps

##### 2.1.3.1 *Peak calling and coverage generation*

The pipeline begins by calling statistically significant coverage peaks from RNA-seq alignments using MACS2, a model-based peak caller originally developed for ChIP-seq but effective for identifying localized enrichment in RNA-seq data. MACS2 models fragment size distributions, calculates local enrichment statistics, and identifies regions exceeding a user-specified false discovery rate (FDR) threshold. In parallel, the pipeline generates normalized coverage tracks for visualization and quality assessment.

##### 2.1.3.2 *Peak processing and selection*

Detected peaks are filtered by MACS2 quality metrics—FDR-adjusted *q*-value and peak score (–10 × log10 of the raw MACS2 *P* value, so higher values reflect stronger enrichment)—and by their overlap with annotated exons/UTRs. For each gene, peaks are ranked by the selected metric (score or *q*-value), and the workflow retains the top-ranked peak or, in multi-peak mode, all peaks. Target windows are trimmed to exon/UTR boundaries (default force_exonic_trimming), ensuring primers are designed within transcribed regions rather than intronic sequence. Peaks failing these thresholds or producing trimmed regions shorter than the minimum requirement are removed and reported in the QC tables for downstream review.

##### 2.1.3.3 *Primer design with Primer3*

Genomic sequences corresponding to selected peak regions are extracted and formatted as input for Primer3. Prior to primer design, the template DNA sequences are corrected for orientation so that all are in the 5${\;}^{\prime }\to$ 3${\;}^{\prime }$ direction. Primer3 then evaluates thermodynamic properties, including melting temperature, GC content, secondary structure propensity, and self-complementarity, to design candidate primer pairs. Because the sequence orientation is standardized in this way, the pipeline selects only left primers (i.e. primers pointing toward the 3${\;}^{\prime }$ end). The pipeline also allows the user to modify the default settings.

##### 2.1.3.4 *Transcriptome specificity screening*

To detect potential off-target binding, candidate primers are aligned to the transcriptome using Bowtie2 v2.5 in sensitive local alignment mode, reporting all valid alignments (-a -L 15 -N 1). This configuration allows one mismatch within the seed and captures suboptimal alignments with additional mismatches outside the seed. For each primer, the pipeline records all alignments, number of mismatches and computes the distance from the alignment position to the corresponding transcript 3${\;}^{\prime }$ end.

##### 2.1.3.5 *Primer selection and optimization*

From the specificity-screened candidates, the pipeline selects optimal primers using a three-stage filtering strategy designed for 3${\;}^{\prime }$-end protocols. In Stage 1 (mismatch filtering), only alignments with a total mismatch count lower or equal to a configurable threshold (default: 3 mismatches) are retained, acknowledging that primers with 1–3 mismatches can still cross-amplify off-target transcripts (Lefever *et al.* 2013). Stage two filters alignments by distance to the transcript 3${\;}^{\prime }$ end, excluding alignments beyond the distance threshold. Stage three requires unique gene mapping: after the prior filtering, surviving primers must align to only one target gene. Primers aligning to multiple genes near their 3${\;}^{\prime }$ ends are rejected to prevent cross-amplification. This filtering prioritizes specificity and protocol compatibility over thermodynamic properties, as PeakPrime has optimized melting temperature and GC content settings during design.

In multi-peak mode an optional isoform-aware optimization step applies a greedy set cover algorithm across all peaks per gene. For each gene, the algorithm iteratively selects the primer that covers the most not yet targeted isoforms, regardless of which peak it originates from. When multiple primers cover equal numbers of new isoforms, ties are broken by selecting the primer closest to the 3${\;}^{\prime }$ end. This strategy ensures maximal isoform coverage while prioritizing biologically relevant primer positions near polyadenylation sites. The process continues until the desired number of primers per gene is reached or all reachable isoforms are covered. In single-peak mode, it simply selects one primer per gene that maximizes distinct isoform coverage.

##### 2.1.3.6 *Visualization and quality control*

**Figure 1:**
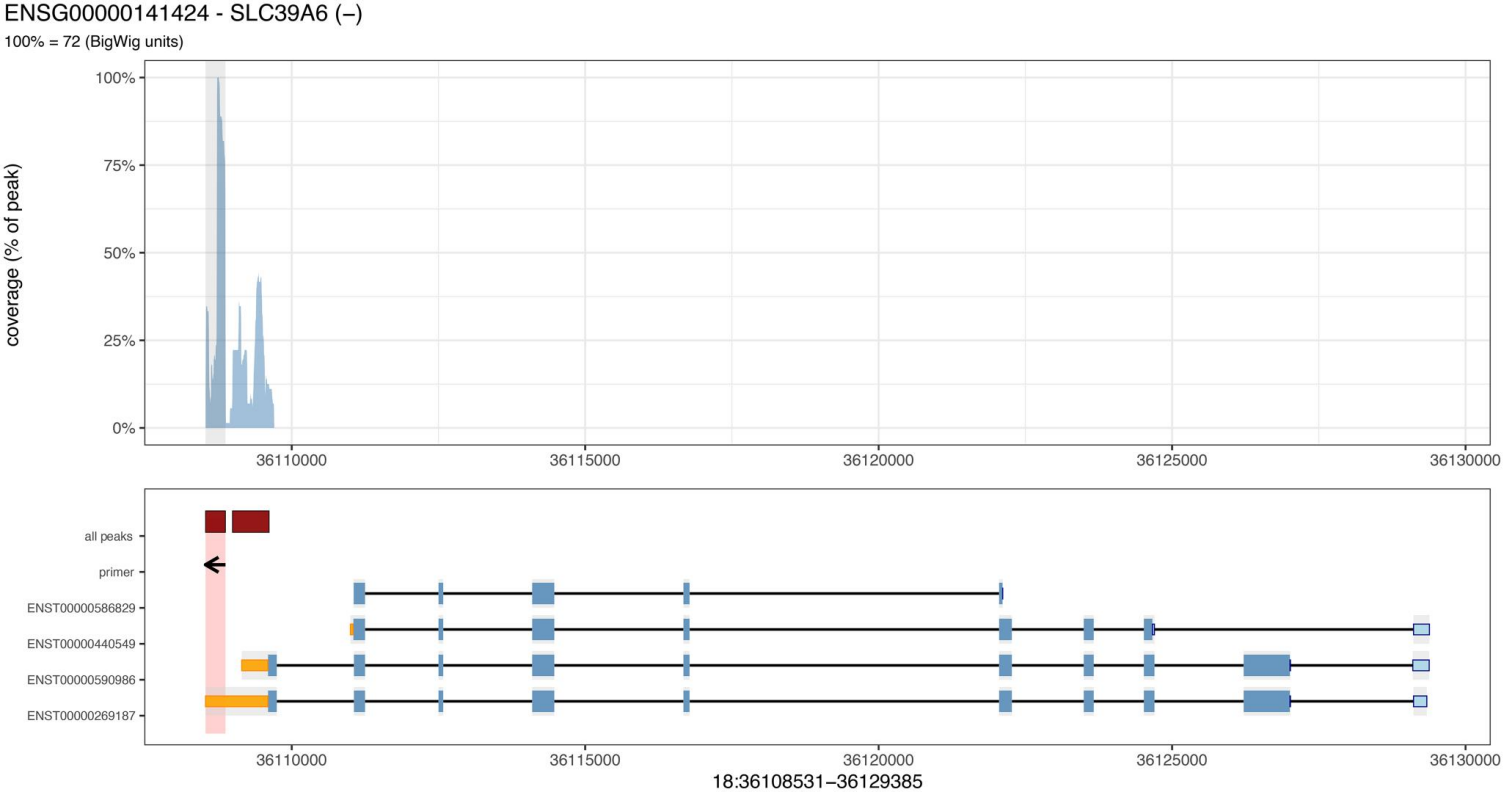
Example visualization showing RNA-seq coverage (top), gene structure with 3${\;}^{\prime }$ UTRs (orange), 5${\;}^{\prime }$ UTRs (light blue), CDS regions (blue), MACS2 called peaks (red) primer direction (arrow) for gene SLC39A6. The selected peak window is highlighted, and all detected peaks within the gene are shown as bars.

When visualization is enabled, the pipeline generates publication-ready plots for each gene showing RNA-seq coverage across the entire gene body, overlaid gene structure with exons for all annotated isoforms, positions of all detected peaks within the gene, the selected target window, and directional indicators for designed primer locations. Quality control metrics including peak significance, exonic overlap, and coverage statistics are annotated on each plot. These visualizations enable rapid assessment of primer design quality and identification of potential issues such as low coverage or complex gene structures (Fig. 1).

#### 2.1.4 Quality control and reporting

Throughout execution, the pipeline generates comprehensive quality control reports documenting the number of genes processed, genes with detected peaks, genes with successfully designed primers, and genes with primers passing specificity screening. For genes failing at any stage, the pipeline records specific failure reasons (insufficient coverage, no significant peaks, sequence complexity preventing primer design, excessive off-target binding) to guide troubleshooting and parameter optimization. Summary statistics report the proportion of primers achieving perfect selectivity, typical isoform coverage, and overall success rates.

#### 2.1.5 Reproducibility and portability

All computational tools are managed through Conda environments with pinned package versions, ensuring consistent behavior across execution platforms. The Nextflow workflow engine provides automatic result caching, enabling interrupted runs to resume from the last successful step without recomputation. Execution logs capture complete provenance information including tool versions, parameter settings, resource usage, and intermediate results, supporting full reproducibility and audit trails for publication.

#### 2.1.6 Performance characteristics

To assess computational scalability, we benchmarked the pipeline across three gene set sizes (50, 100, and 200 genes) using a standardized 10M-read subset of the human neuroblastoma cell line IMR-32 random-primed RNA dataset (Tables 1–3, available as supplementary data at *Bioinformatics Advances* online). All runs completed successfully with 100% task completion rates (12/12 tasks) on AMD EPYC processors. Total execution time scaled sub-linearly with gene count (7 m 24 s for 50 genes, 11 m 57 s for 100 genes, 13 m 44 s for 200 genes), demonstrating efficient parallelization and minimal overhead. Notably, the average per-gene processing time decreased with larger datasets (8.88 s/gene for 50 genes vs. 4.12 s/gene for 200 genes), indicating that fixed-cost processes (MACS2 peak calling: 16 seconds constant across all runs; BAM indexing: <2 seconds) dominate execution time for small datasets but amortize efficiently at scale (Table 1 and Fig. 1, available as supplementary data at *Bioinformatics Advances* online).

Peak memory consumption remained constant at 3.47 GB across all gene set sizes, consistently occurring during the PROCESS_MACS2_PEAKS step (85% of configured 4 GB allocation). This memory profile demonstrates that peak usage is determined by genome annotation processing rather than gene set size, making the pipeline highly memory-efficient even for large-scale analyses. Disk I/O scaled modestly with gene count (8.2 GB read for 50 genes to 11.6 GB read for 200 genes), while disk writes remained constant at 672 MB, reflecting the dominance of read operations during transcript mapping and peak annotation (Table 2 and Fig. 1, available as supplementary data at *Bioinformatics Advances* online).

**Figure 2:**
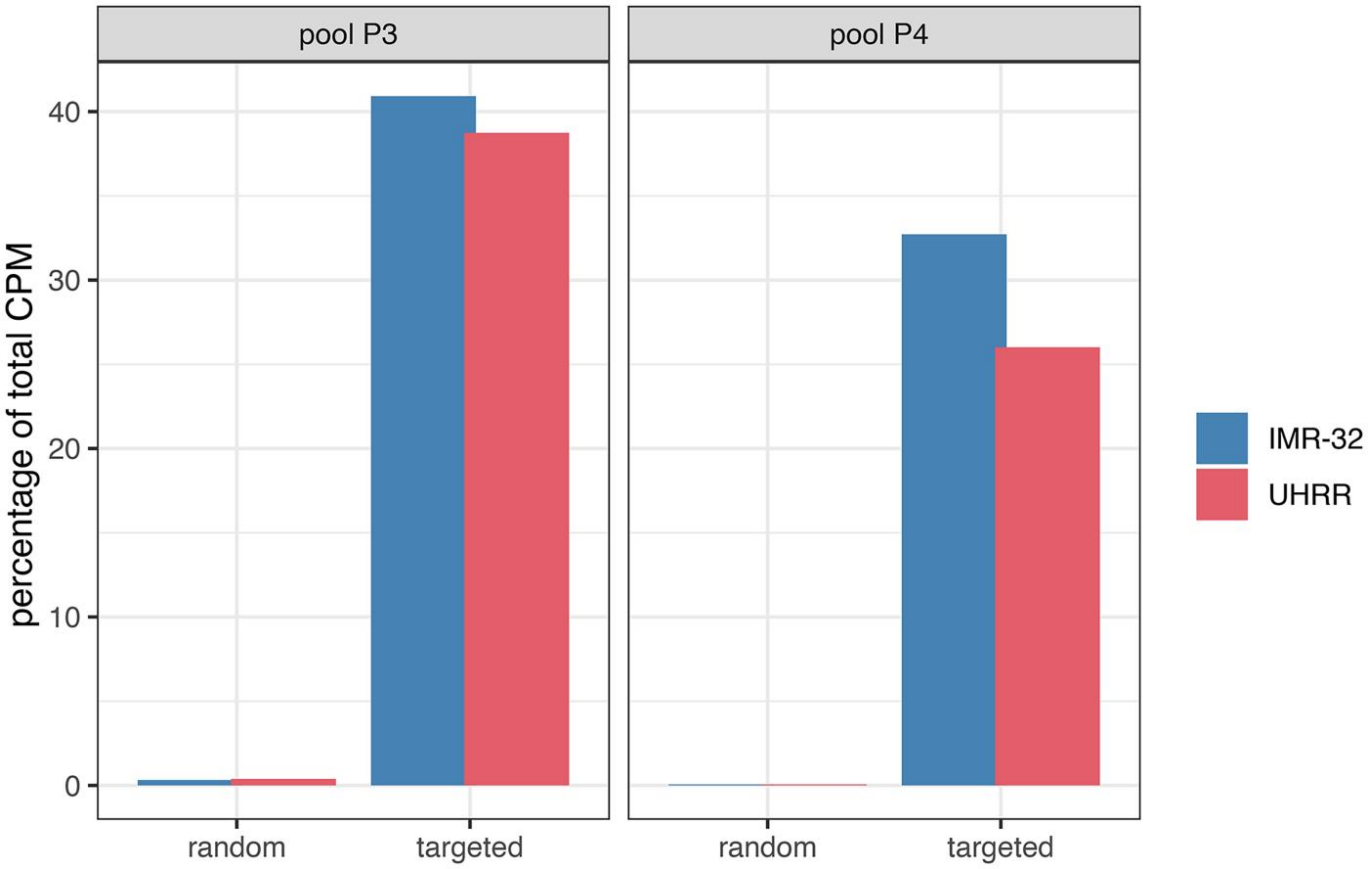
Target gene capture efficiency for each primer pool. Bar plots comparing the percentage of total CPM counts attributable to the targeted genes in random-primed and targeted libraries.

Process-level timing analysis revealed distinct scaling behaviors: gene-dependent processes (PROCESS_MACS2_PEAKS, OPTIMIZE_MULTIPEAK) scaled linearly (168 s → 328 s for peak processing; 19 s → 51 s for optimization), primer-dependent processes (RUN_PRIMER3, ALIGN_PRIMERS) exhibited sub-linear scaling due to primer generation limits (167 s → 305 s for primer3; 8 s → 32 s for alignment), and dataset-dependent processes (MACS2_CALLPEAK, BAM_INDEX) remained constant regardless of gene count (Table 3 and Fig. 2, available as supplementary data at *Bioinformatics Advances* online). Based on these metrics, we recommend 4–5 GB RAM for datasets ≤200 genes, 10–15 GB disk space, and single-core execution, as the pipeline achieves linear scalability without requiring extensive computational resources. For large-scale analyses (>500 genes), the constant-time overhead becomes negligible (<5% of total runtime), making the pipeline well-suited for high-throughput primer design applications.

### 2.2 Selection of target genes

**Figure 3:**
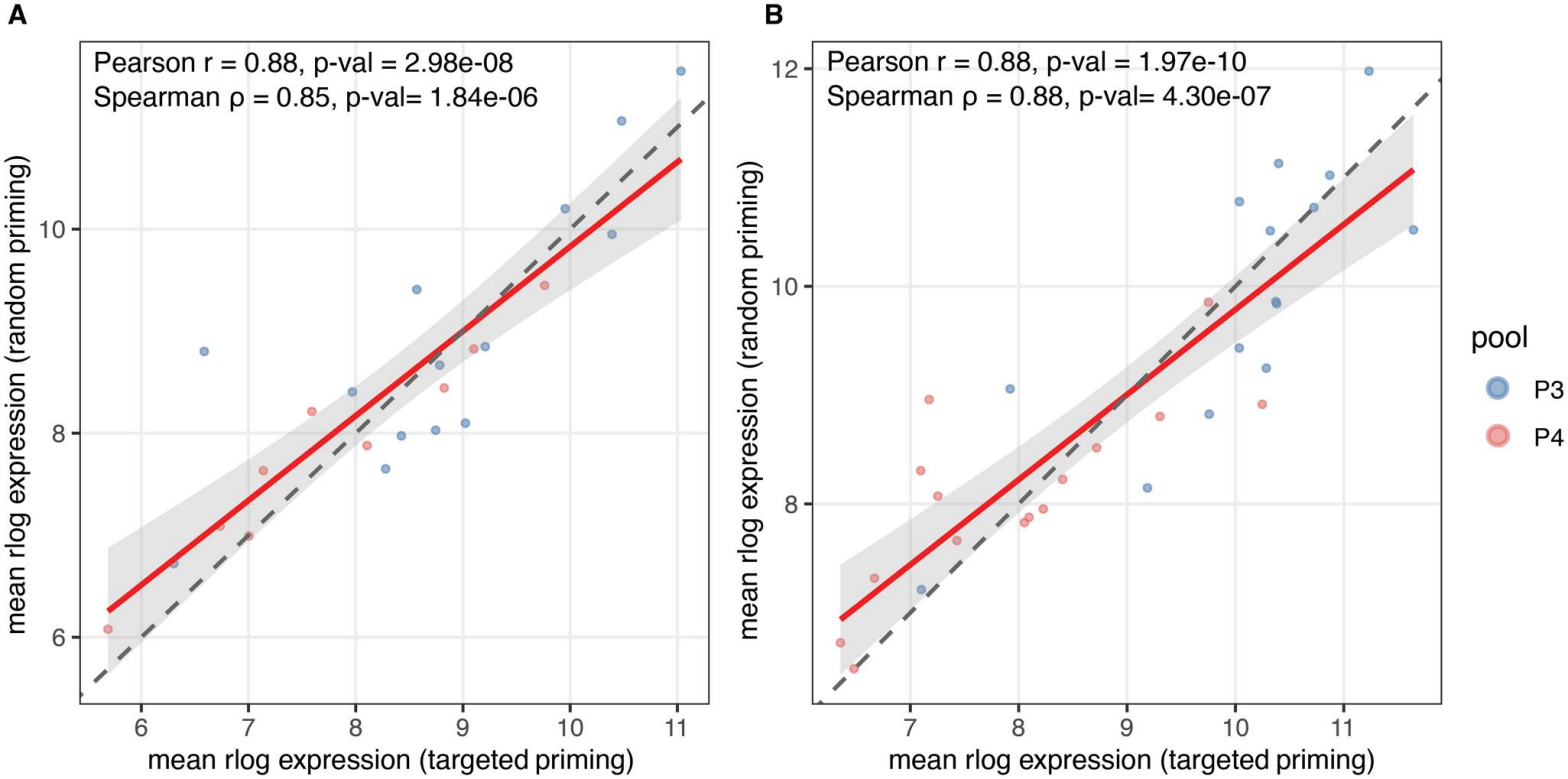
Correlation of gene-level expression between random-primed and targeted 3${\;}^{\prime }$ RNA-seq. Scatter plots of rlog-transformed mean counts per gene comparing random-primed 3${\;}^{\prime }$ RNA-seq (*x*-axis) to PeakPrime-targeted libraries (*y*-axis). (A) Universal Human Reference RNA (UHRR; *n = *23 genes). (B) IMR-32 neuroblastoma cell line derived RNA (*n = *30 genes).

Gene counts derived from whole-transcriptome 3${\;}^{\prime }$ RNA-seq of the human neuroblastoma cell line IMR-32 were used to randomly select 30 genes in the lower half of the cumulative CPM curve (Fig. 3 and Table 4, available as supplementary data at *Bioinformatics Advances* online). The data was generated with a custom bulk 3${\;}^{\prime }$ protocol that employs random priming during second-strand synthesis (Section 2.4). Specifically, the two IMR-32 samples described in Section 2.3 were processed with this custom protocol to produce random-primed 3${\;}^{\prime }$ RNA-seq libraries; the resulting alignments provided the empirical 3${\;}^{\prime }$ coverage profiles used by PeakPrime to identify high-confidence 3${\;}^{\prime }$ peaks and to define exonic windows for primer design. Sequencing-based validation in this study focused on the selected 30 genes, where gains in sensitivity are expected to be pronounced due to their low abundance in the random-primed whole transcriptome data.

### 2.3 Samples and RNA reference material

Universal Human Reference RNA (UHRR, Agilent) and total RNA from the human neuroblastoma cell line IMR-32 were used as input materials. RNA integrity was assessed on a 5300 Fragment Analyzer (Agilent). For random-primed 3${\;}^{\prime }$ RNA-seq, two independent UHRR libraries and two independent IMR-32 libraries were prepared. For targeted 3${\;}^{\prime }$ RNA-seq, two UHRR libraries and two IMR-32 libraries were prepared using two primer pools (P3 and P4), each containing 15 primers, with two technical replicates per pool.

### 2.4 Custom 3${\;}^{\prime }$ end library preparation

For the random-primed libraries, a custom bulk 3${\;}^{\prime }$ RNA-seq library preparation protocol (Van Droogenbroeck *et al.*, unpublished results) was used. Briefly, polyadenylated transcripts were reverse transcribed using a sample-barcoded oligo(dT)-VN primer which consist of a Nextera Read 1 sequence, a 10-nucleotide unique molecular identifier (UMI), a 36-nucleotide barcode, 18 T nucleotides followed by a degenerate VN anchor. Second-strand synthesis (Klenow Fragment 3${\;}^{\prime }$>5${\;}^{\prime }$ exo-) was performed using a tailed random primer to enable amplification across captured cDNAs. Libraries were PCR-amplified, purified, and quantified prior to sequencing. All enzymatic reaction conditions, incubation times, and cleanup ratios are recorded in the wet-lab SOP and will be disclosed upon publication of the protocol.

Key features of the protocol include:

- Oligo(dT)-VN capture to prime near the poly(A) junction.
- Embedded sample-specific barcode within the oligo(dT) primer for early multiplexing
- Random-primed second-strand synthesis to generate double-stranded cDNA for library amplification
- Library amplification using PrimeTime DNA polymerase (IDT; 1055772); bead-based purification.

For whole transcriptome libraries, first-strand cDNA was synthesized in 100 μL using 30 nM oligo(dT)-VN capture probe, 1× Protoscript RT buffer (NEB; M0368X), 10 ng/μL RNA, 0.01 M DTT (NEB; M0368X), 0.5 mM dNTPs, 10 U/μL Protoscript RT (NEB; M0368X), 0.4 U/μL RnaseOUT (Invitrogen; 10154652), and 2 μL of 1:100 ERCC ExFold mix 1 (Invitrogen; 4456739).

For targeted libraries, first-strand cDNA was performed in 120 μL using 3 nM oligo(dT)-VN capture probe, 1× Protoscript RT buffer (NEB; M0368X), 10 ng/μL RNA, 0.01 M DTT (NEB; M0368X), 0.5 mM dNTPs, 3 U/μL Potoscript RT (NEB; M0368X), and 0.4 U/μL RnaseOUT (Invitrogen; 10154652).

The 10 μL of both first-strand cDNA products underwent 0.8× (whole transcriptome) or 0.9× (targeted) bead-cleanup with AMPure XP beads (Beckman Coulter) and elution in 8 μL.

Second-strand cDNA synthesis was performed using 50 mM randomer, 0.5 μM dNTPs, 1× NEBuffer2 (NEB; M0212S), and 0.25 U/μL Klenow exo- (NEB; M0212S). For targeted libraries, the random primer was replaced with a pool of PeakPrime-designed primers, each at 1 nM (final reaction), combined with a Nextera-compatible universal primer at 200 nM using 1× PrimeTime master mix (IDT; 1055772). Targeted pre-amplification was performed for five PCR cycles in a total reaction volume of 20 using an initial 3 min enzyme activation step at 95°C followed by five cycles of 15 min at 95°C and 1 min at 60°C, and finishing with 2 min at 72°C.

After second strand cDNA synthesis or pre-amplification, samples underwent bead cleanup with a 0.8× (whole transcriptome) or 0.9× (targeted) ratio AMPure XP beads (Beckman Coulter) and were eluted in a 10 μL volume; 1 μL (of each eluted 10 μL sample) was used as input in a 5 μL qPCR reaction (to determine optimal number of indexing PCR cycles), using 1× PrimeTime master mix, final 1/40 diluted EvaGreen (VWR; 31000-T) and 250 nM of each Nextera compatible indexing primer. PCR was run for 45 cycles on a LightCycler 480 (Roche) using the same cycling protocol as mentioned above. The optimal cycle number was defined as the rounded Cq-value of the library. The remaining 9 μL pre-amplified library was then indexed (250 nM indexing primer concentration and 1× PrimeTime mastermix) for the optimal number of cycles in a total reaction volume of 22.5 μL and finalized with a 0.7× (whole transcriptome) or 0.9× (targeted) ratio bead cleanup Ampure XP beads (Beckman Coulter), eluted in a final 13 μL volume. Libraries were equimolarly pooled to the lowest concentrated sample based on KAPA qPCR quantification (Roche; KK4854) and average fragment length between 200 and 1450 bp according to Fragment Analyzer.

### 2.5 Sequencing

Libraries were quality-checked (Fragment Analyzer) and quantified using the Qubit dsDNA HS Assay Kit (Thermo Fisher Scientific; 10606433) prior to sequencing on a NextSeq 2000 (Illumina). Loading concentration was 700 pM (with 10% PhiX spike-in). A summary of the sequencing depth and read length is shown in Table 5, available as supplementary data at *Bioinformatics Advances* online.

### 2.6 Read processing and gene-level quantification

Basecalling and demultiplexing were performed with bcl-convert v4.3.13. Sequencing reads were processed using a pipeline implemented in Nextflow v25.04 available at https://github.com/OncoRNALab/QSP_nextflow.git.

Read 1 was parsed to extract sample barcodes and unique molecular identifiers (UMIs) using umi_tools v1.1.4. Adapter sequences, residual primer sequences and terminal poly(A) tails were trimmed from read 2 using cutadapt v4.5. Filtered reads were aligned to the human reference genome (GRCh38) using STAR v2.7.8a with settings tailored to short, 3${\;}^{\prime }$-biased reads. Alignments were sorted and indexed using samtools v1.16. PCR and technical duplicates were removed with umi_tools dedup, using both UMI sequences and mapping coordinates. Gene-level counts were obtained using featureCounts from the Subread package with an Ensembl GTF annotation, counting uniquely aligned reads overlapping exonic regions. Per-sample count tables were merged into a single gene-by-sample matrix using custom R and Python scripts. Quality control included FastQC v0.11.9, MultiQC (latest version) and additional metrics such as library complexity, fraction of reads assigned to genes and 3${\;}^{\prime }$-end bias, all computed within the Nextflow workflow. Containerized tools were generated with Singularity v1.4.2.

### 2.7 Parameter optimization

Peak calling and primer design parameters were tuned on the custom 3${\;}^{\prime }$ RNA-seq dataset to maximize design success rate and predicted selectivity while maintaining proximity to 3${\;}^{\prime }$ peaks. Explored hyperparameters included MACS2 q-value/score thresholds, minimum exonic overlap, design-window size, and Primer3 constraints (Tm, delta-Tm, GC). Selected settings balanced design success rate (fraction of targets with at least one design) and selectivity (in silico off-target risk).

### 2.8 Empirical qPCR validation of primers

To evaluate 3${\;}^{\prime }$ end primer performance, we used PeakPrime to design primers within a fixed distance from the transcript 3${\;}^{\prime }$ end. As input, the pipeline accepts either a list of Ensembl gene identifiers or a FASTA file containing transcript sequences. When Ensembl identifiers are provided, the corresponding MANE transcripts are retrieved and used to obtain the reference sequences. In both cases, the pipeline extracts a user-defined number of bases from the 3${\;}^{\prime }$ end of each transcript and passes this subsequence to Primer3 for primer design. To evaluate this distance-based design mode, we used External RNA Control Consortium (ERCC) ExFold mix 1 spike-in controls (Invitrogen; 4456739) as targets. Primers were designed within 40–200 nucleotides upstream of the 3${\;}^{\prime }$ end for 40 unique ERCCs (randomly selected across all concentration levels). For qPCR quantification of ERCC ExFold mix 1 RNA, the same first-strand cDNA synthesis method as for whole transcriptome libraries was used. This first-strand cDNA was directly used as input in a 5 μL qPCR reaction using 1× PrimeTime, final 1/40 dilution of EvaGreen, and 250 nM of each PeakPrime designed ERCC primer in combination with a Nextera compatible primer for 45 cycles using the cycling protocol mentioned in Section 2.4.

### 2.9 Statistical analysis

All statistical analyses were conducted in R v4.5.1. To avoid spurious correlations driven by low counts, genes with a mean count below 10 across the samples in a given comparison were excluded. Normalization and variance-stabilising transformation were performed using the regularized logarithm (rlog) function from DESeq2 v1.5.0. For each RNA type (UHRR or IMR-32) and library type (random or targeted), mean rlog expression values were calculated for each gene across technical replicates. Pearson and Spearman correlation coefficients were then computed between random-primed and targeted mean rlog counts.

Similarly, to assess differential expression between UHRR and IMR-32, DESeq2 was used to estimate log2 fold changes for each gene within each library type. For the 30 gene panel, log2 fold changes from random-primed libraries were compared to those from targeted libraries using linear regression and correlation analysis. All scripts used to perform these analyses and generate the figures are provided in the PeakPrime repository.

## 3 Results

### 3.1 Overview of primer design and validation strategy

We developed PeakPrime, a Nextflow-based pipeline that performs coverage-informed primer design from 3${\;}^{\prime }$ RNA-seq data and applied it to generate a targeted primer panel for low-abundance transcripts. Using random-primed IMR-32 3${\;}^{\prime }$ RNA-seq data, 30 genes were selected from the 50% lowest abundant genes. PeakPrime identified enriched 3${\;}^{\prime }$ coverage peaks for these genes, designed strand-appropriate primers within exonic windows around the peaks, and filtered them for selectivitity against the human transcriptome. To minimize amplification bias, PeakPrime applies strict Primer3 constraints on amplicon length (40–1000 nt), melting temperature (59–61°C), GC content (35%–65%), and self-complementarity, reducing variability in PCR efficiency across targets. For the experiments reported here, primers were further chosen to produce amplicons shorter than 300 nt, enhancing amplification uniformity and compatibility with downstream analyses. The 30 primers were synthesized in two pools of 15 primers each (pool P3 containing 15 targets in the 50%–75%-tile abundance and pool P4 containing targets in the lowest quartile) and incorporated into the pre-amplification step of the targeted 3${\;}^{\prime }$ RNA-seq protocol. Targeted 3${\;}^{\prime }$ RNA-seq libraries were then prepared for both UHRR and IMR-32 and compared with random-primed controls to assess preservation of relative expression and potential gains in detection of low-abundance genes.

### 3.2 Enrichment of low-abundant gene counts

Because the primer panel was deliberately designed for low-abundance targets, we first evaluated detection performance by examining the percentage of total CPM counts attributable to the targeted genes in both pools. For the targeted genes in pool P3, CPM counts corresponded to 0.31%–0.38% of the total counts in random-primed libraries, whereas they ranged between 38.74% and 40.92% in the targeted libraries, an enrichment of 115-fold. A similar pattern was observed for genes in pool P4, with CPM counts 0.6% of the total counts in random-primed libraries, but between 26.01% and 32.72% in the targeted libraries (49-fold) (Fig. 2).

### 3.3 Targeted 3${\;}^{\prime }$ RNA-seq preserves relative abundance

Next, we validated whether targeted priming preserves relative gene expression levels measured by random-primed whole transcriptome 3${\;}^{\prime }$ RNA-seq. For each condition, rlog-transformed counts were averaged across technical replicates and compared between random-primed and targeted libraries for the 30-gene panel.

As expected for IMR-32, all 30 genes passed the low-count filter and correlations between random and targeted libraries were again strong (Pearson *r* = 0.88; *P* val < 1e−4; Spearman ρ = 0.88; *P* val < 1e−4) (Fig. 3). For UHRR, 14 genes from P3 pool and 9 genes from P4 pool passed the low-count filter (mean count ≥10). The correlation between random-primed and targeted mean rlog counts was high (Pearson *r* = 0.88; *P* val < 1e−4; Spearman ρ = 0.85; *P* val < 1e−4) (Fig. 3).

These results indicate that targeted priming recapitulates the rank ordering and relative abundance of low-abundant genes observed with random priming across both neuroblastoma cell-line RNA (used to select the genes) and universal human reference RNA.

### 3.4 Targeted priming recapitulates log2 fold changes between UHRR and IMR-32

We next evaluated whether targeted priming reproduces the relative differences in gene expression between UHRR and IMR-32 that are observed with random priming in the whole transcriptome data. For each method, DESeq2 was used to estimate log2 fold changes between UHRR and IMR-32 for the 30-gene panel, and these log2 fold changes were compared between methods.

**Figure 4:**
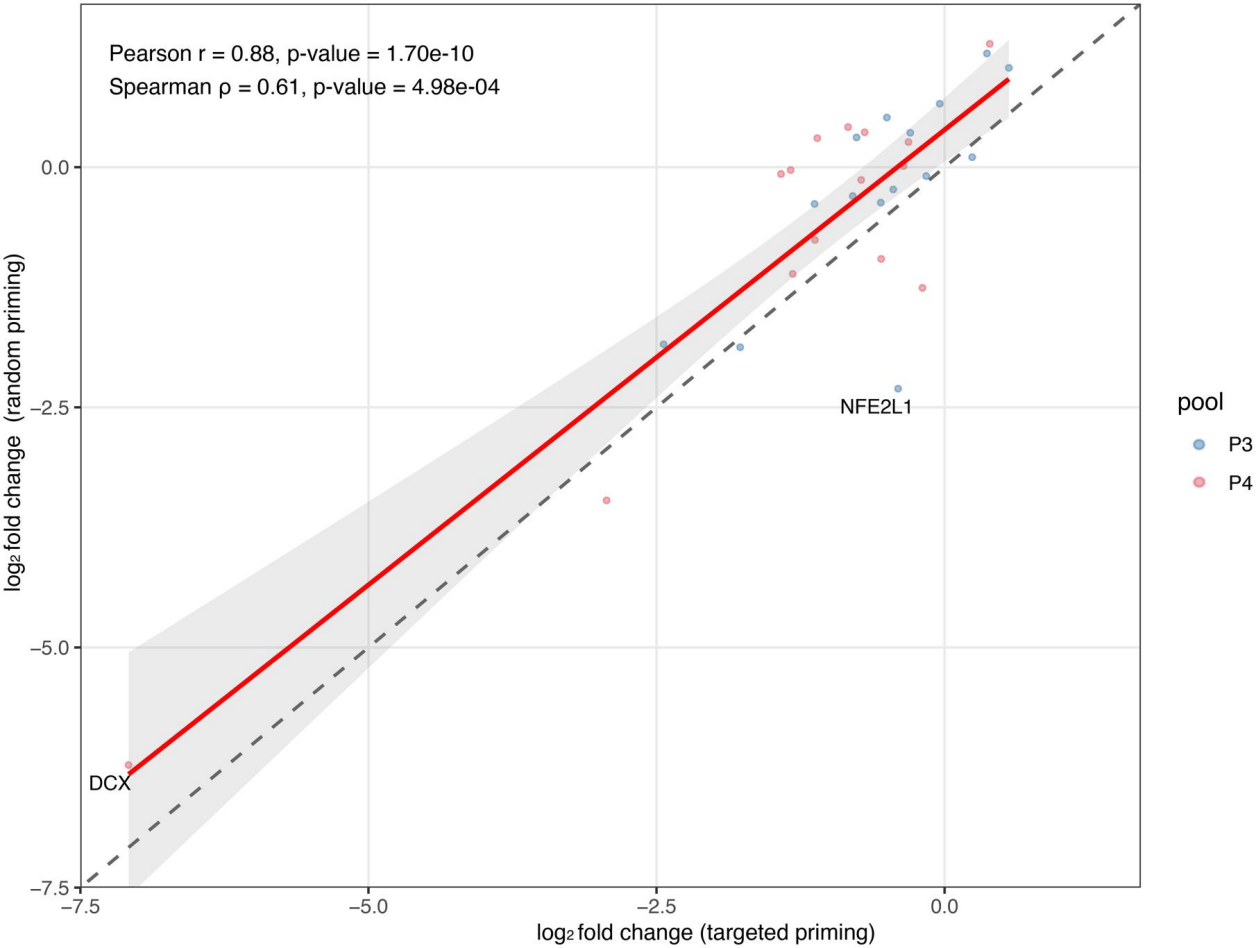
Correlation of UHRR–IMR-32 log2 fold changes between random-primed and targeted 3${\;}^{\prime }$ RNA-seq. Scatter plots comparing DESeq2 ${\;\text{log}\;}_{2}$ fold changes (UHRR vs. IMR-32) obtained from random-primed libraries (*x*-axis) to those from PeakPrime-targeted libraries (*y*-axis) for the 30 targeted genes.

The log_2 fold changes estimated from targeted and random-primed libraries were globally concordant, with a high Pearson correlation (*r* = 0.88; *P* val < 1e−4) indicating that effect sizes between UHRR and IMR-32 are largely preserved across protocols, despite a more modest Spearman correlation (ρ = 0.61; *P* val < 1e−3) that reflects some rank reordering driven by a small number of discordant genes (Fig. 4). Inspection of individual genes revealed no consistent log2 fold changes directional bias; instead, deviations were gene-specific, with both over- and under-estimation observed relative to random priming. Two genes emerged as clear outliers in this comparison. DCX, a neuroblastoma-associated marker (Afanasyeva *et al.* 2021), showed an extreme upregulation (log_2FC > 6) in IMR-32 relative to UHRR, substantially higher than the rest of the panel; this apparent difference is caused by DESeq2 handling of absent counts in UHRR. In contrast, NFE2L1, another gene expected to be elevated in neuroblastoma (De Preter *et al.* 2006), displayed a log_2FC > 2 only in the random-primed libraries, suggesting protocol-specific quantification differences, for example due to isoform-dependent coverage, or alternative polyadenylation events that are captured by random priming but only partially represented by the single 3${\;}^{\prime }$-anchored primer used in the targeted assay. Together, these results indicate that while targeted 3${\;}^{\prime }$ RNA-seq recapitulates the overall differential expression patterns obtained with random priming, the targeted approach could benefit from choosing more than one primer per gene to capture more isoforms.

### 3.5 Distance-based 3${\;}^{\prime }$ primer design shows quantitative performance on ERCC spike-ins

**Figure 5:**
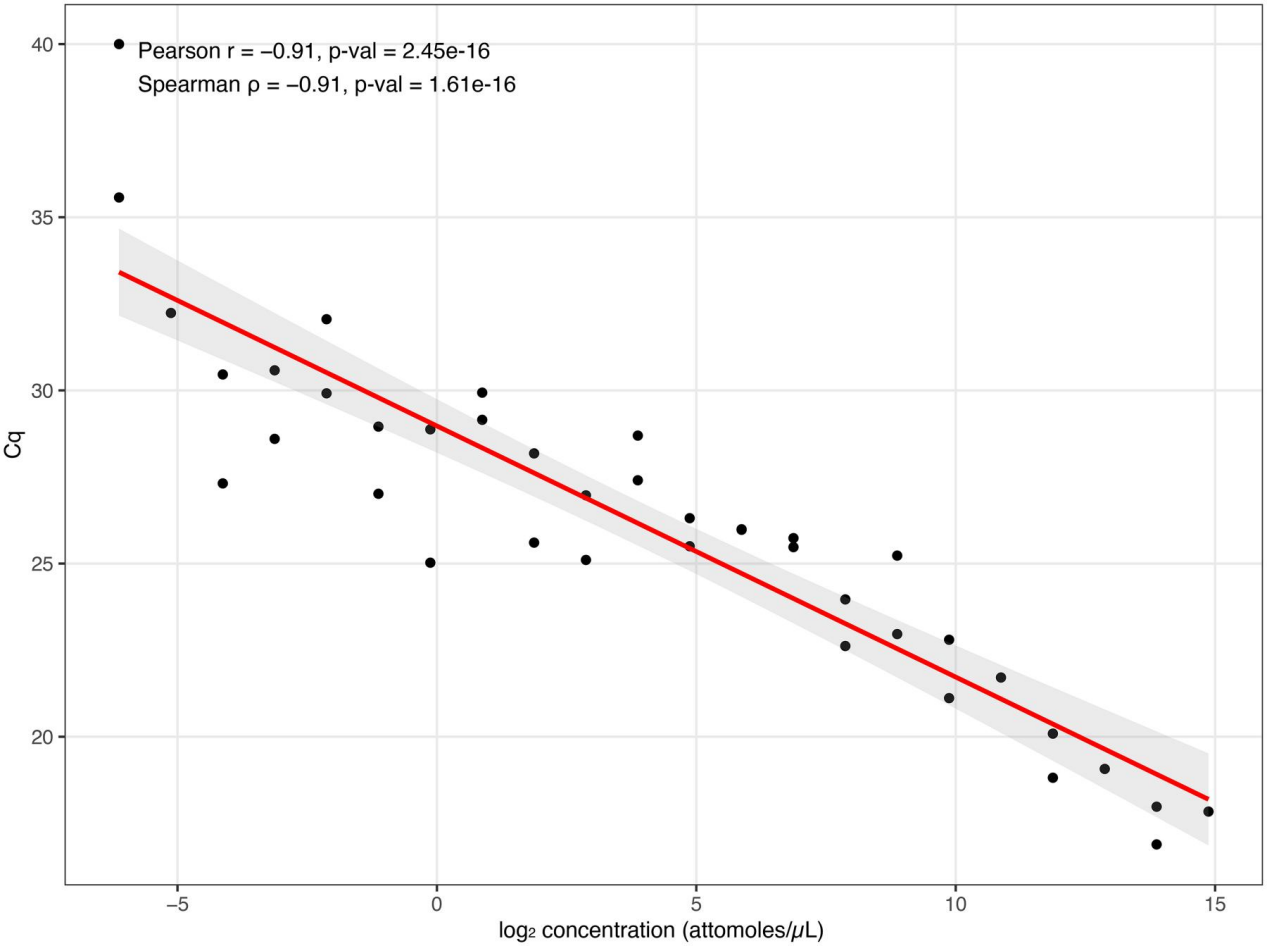
Correlation between ERCC spike-in concentration and qPCR Cq values for distance-based 3${\;}^{\prime }$ primer design. Cq values for 40 ERCC transcripts (*y*-axis) are plotted against their known ${\;\text{log}\;}_{2}$ input concentrations (*x*-axis). The line shows the linear regression fit.

Using the distance-based design mode, we successfully generated primer pairs for 40 distinct ERCC spike-in transcripts with a 3${\;}^{\prime }$-proximal target region 200 nucleotides from the transcript end. All primers produced quantifiable Cq values under the chosen qPCR conditions. When plotted against the known log_2 input concentrations of the ERCC mix, the Cq values exhibited a strong linear relationship (Pearson *r* = −0.91; *P* val < 1e−4; Spearman ρ = −0.91; *P* val < 1e−4) (Fig. 5). This correlation indicates that primers designed from short 3${\;}^{\prime }$ terminal windows using the pipeline maintain quantitative behavior over the 21 log2 units dynamic range of the ERCC spike-in panel. These primers could be used to quantify spike-in RNA in future targeted 3${\;}^{\prime }$ end RNA-seq experiments, serving as quality control.

## 4 Conclusions

Together, these analyses demonstrate that PeakPrime-guided targeted 3${\;}^{\prime }$ RNA-seq is a useful complement to random-primed 3${\;}^{\prime }$ protocols for quantifying predefined panels of low-abundance genes. The method maintains concordant expression estimates and differential expression patterns between UHRR and IMR-32, while substantially increasing the fraction of reads assigned to the selected targets and improving detection of the lowest-abundance genes. As with other targeted sequencing approaches, primer-specific enrichment efficiencies may differ among targets, which can affect absolute comparisons of abundance levels across targets. PeakPrime is therefore primarily intended for robust gene-level quantification and differential expression analyses rather than absolute cross-gene expression comparisons without the use of spike-in controls or other external calibration standards. At the same time, PeakPrime provides a flexible framework for scaling coverage-informed primer design to larger panels and additional assay formats, and its efficient implementation enables practical use on standard workstations and in high-performance computing environments.

In this study, target genes were selected solely on the basis of their transcript abundance, using CPM-derived expression classes from whole transcriptome 3${\;}^{\prime }$ RNA-seq data. However, PeakPrime is agnostic to how gene panels are defined and can be combined with external feature-selection methods tailored to specific experimental contexts. For example, in single-cell RNA-seq studies, genes could be chosen using tools such as scPNMF or related approaches that identify informative or biologically relevant gene sets, and these genes could then be passed to PeakPrime for coverage-informed primer design. This decoupling of gene selection from primer design facilitates the use of PeakPrime for diverse applications, including focused panels derived from bulk, single-cell, or spatial transcriptomics data.

In the present work, a single primer per gene was selected on the basis of the top-ranked 3${\;}^{\prime }$-end coverage peak, which typically reflects the dominant isoform observed in the random-primed datasets. This design choice implies that targeted libraries primarily capture the predominant isoform for each gene, whereas random priming can sample multiple isoforms arising from alternative splicing or alternative polyadenylation. As a consequence, minor isoforms that contribute to the gene-level signal in the random-primed data may be under-represented or not captured at all in the targeted libraries, potentially introducing isoform-dependent biases into some of our comparisons. The PeakPrime pipeline includes an alternative design mode in which primers are generated for all high-confidence peaks of a given gene and an optimization algorithm selects combinations of primers that jointly cover the largest possible set of isoforms. Although this multi-primer, multi-peak mode was not used in the current study, future applications aimed at isoform-resolved quantification or at genes with complex 3${\;}^{\prime }$-end architecture would benefit from exploiting this extended design mode, ideally in combination with data-driven gene selection strategies, to minimize isoform bias while retaining the sensitivity gains of targeted 3${\;}^{\prime }$ RNA-seq.

## Supplementary Material

vbag080_Supplementary_Data

## Data Availability

PeakPrime source code, documentation, and guides can be accessed at https://github.com/OncoRNALab/PeakPrime. The software is released under the PolyForm Noncommercial 1.0.0 license, which permits reuse and modification for non-commercial purposes under the terms of the license. The raw fastq files generated in this study have been deposited in the Zenodo repository (DOI: 10.5281/zenodo.17821004). Processed gene count data, as well as all scripts required to reproduce the analyses and figures presented in this manuscript, are available in the PeakPrime GitHub repository under the “manuscript” branch. The pipeline to process raw fastq files can be accessed at https://github.com/OncoRNALab/QSP_nextflow.git
