# Supplementary material for: PeakPrime: a peak-guided primer design pipeline for target enrichment in 3′-end RNA-seq
Source: Bioinform Adv. 2026 Mar 19;6(1):vbag080. doi: 10.1093/bioadv/vbag080 (PMC13034549; doi:10.1093/bioadv/vbag080)
Supplement: vbag080_Supplementary_Data [file vbag080_supplementary_data.zip › Supplementary Information.docx]

# **Supplementary Information**

**Supplementary Table 1:** Comparative scalability analysis: pipeline performance across gene set zizes. overall execution metrics.

| gene set ize | execution duration | CPU-hours | tasks completed | avg time per gene | success rate |
| --- | --- | --- | --- | --- | --- |
| 200 genes | 13 m 44 s | 0.2 | 12/12 | 4.12 s/gene | 100% |
| 100 genes | 11 m 57 s | 0.2 | 12/12 | 7.17 s/gene | 100% |
| 50 genes | 7 m 24 s | 0.1 | 12/12 | 8.88 s/gene | 100% |

**Supplementary Table 2:** Comparative scalability analysis: peak resource usage by gene set size

| gene set size | meak memory (GB) | peak memory process | disk read (GB) | disk write (MB) |
| --- | --- | --- | --- | --- |
| 200 genes | 3.47 | PROCESS_MACS2_PEAKS | 11.64 | 673 |
| 100 genes | 3.47 | PROCESS_MACS2_PEAKS | 9.36 | 673 |
| 50 genes | 3.47 | PROCESS_MACS2_PEAKS | 8.23 | 672 |

**Supplementary Table 3:** Comparative scalability analysis: key process execution time (seconds)

| process | 200 genes | 100 genes | 50 genes | scaling pattern |
| --- | --- | --- | --- | --- |
| MACS2_CALLPEAK | 15.1 | 16.6 | 16.0 | constant (~16 s) |
| PROCESS_MACS2_PEAKS | 328.1 | 245.1 | 168.0 | linear with genes |
| RUN_PRIMER3 | 304.6 | 306.2 | 167.1 | sub-linear |
| ALIGN_PRIMERS | 31.6 | 26.6 | 7.7 | linear with primers |
| OPTIMIZE_MULTIPEAK | 51.0 | 37.1 | 18.7 | linear with genes |

**Supplemetary Table 4.** Ensembl gene ID, HUGO gene symbol, primer sequence designed with PeakPrime and library preparation pool

| gene ID | HUGO gene symbol | primer sequence | pool |
| --- | --- | --- | --- |
| ENSG00000108960 | MMD | ACACCAACCAAACCACTCCT | C3 |
| ENSG00000133612 | AGAP3 | CTGCCCTAAGGTGCCATTGA | C3 |
| ENSG00000141424 | SLC39A6 | ACCAGACTGGGTTATTGCCA | C3 |
| ENSG00000082641 | NFE2L1 | GTGTGAACCTGGGAGTGCTT | C3 |
| ENSG00000163902 | RPN1 | GCAGTGTGAGTTTGCCGTTT | C3 |
| ENSG00000112078 | KCTD20 | TTCAGTGCCACTTCTTGCCA | C3 |
| ENSG00000142192 | APP | TAACCCCGGGCAAGACTTTT | C3 |
| ENSG00000101367 | MAPRE1 | AGTGCATGCTCTGTGTGGAA | C3 |
| ENSG00000125458 | NT5C | GAGTGGAGCAGGCAGATACC | C3 |
| ENSG00000156298 | TSPAN7 | GCCTCTGTCTAGCATGCCAA | C3 |
| ENSG00000159023 | EPB41 | CTCGGGCAGCAGAATCAGAT | C3 |
| ENSG00000179950 | PUF60 | GTGGCTGAAGTGTACGACCA | C3 |
| ENSG00000162607 | USP1 | AACCTGGACAGTTCGCTTCT | C3 |
| ENSG00000143641 | GALNT2 | GCGTGCCGAAGTTAGTTGTC | C3 |
| ENSG00000225663 | MCRIP1 | TCCCTTTTCCTTCCTGTGCC | C3 |
| ENSG00000165948 | IFI27L1 | GGAGATGACTTTCCTGGGCC | C4 |
| ENSG00000130312 | MRPL34 | CCCAGTACCCTGACTACCGA | C4 |
| ENSG00000140829 | DHX38 | TGTATGCGTGACTTGGCTGT | C4 |
| ENSG00000196876 | SCN8A | CACCTCCTCGACTTGTTCCC | C4 |
| ENSG00000247596 | TWF2 | GCACAGCCCAGAAACCTTTG | C4 |
| ENSG00000167548 | KMT2D | TTTAAAGCGCCCTCTCTCCC | C4 |
| ENSG00000068383 | INPP5A | AGCTGTTGGTCGTTCTGAGG | C4 |
| ENSG00000123384 | LRP1 | AAACATTCCTCCAGCCTCCC | C4 |
| ENSG00000117000 | RLF | ACCTGAGGGTGCTTTGTGTA | C4 |
| ENSG00000142528 | ZNF473 | GAAAACTGGCAGCCATTGGG | C4 |
| ENSG00000213015 | ZNF580 | AGCCTGTGCAAGTGACATGA | C4 |
| ENSG00000077279 | DCX | CGCAGCATTCAATACCAGGC | C4 |
| ENSG00000185187 | SIGIRR | GATCTCGGCTCGCGAAACTA | C4 |
| ENSG00000140521 | POLG | GCCAACATCGGTGTGGACTA | C4 |
| ENSG00000136100 | VPS36 | AGGGCCTGGACACAATAAGC | C4 |

**Supplementary Table 5.** Sequencing depth and read length for all samples included in the experiment

| Sample ID | Library type | Sample type | number of reads | Read length | instrument |
| --- | --- | --- | --- | --- | --- |
| RNA028684 | random priming | UHRR | 4309679 | 51-51 | NextSeq 2000 |
| RNA028685 | random priming | UHRR | 3080920 | 51-51 | NextSeq 2000 |
| RNA033258 | random priming | IMR-32 | 55812962 | 47-151 | NextSeq 2000 |
| RNA033259 | random priming | IMR-32 | 47295239 | 47-151 | NextSeq 2000 |
| RNA033944 | targeted | UHRR_C3_1nM | 662318 | 47-54 | NextSeq 2000 |
| RNA033945 | targeted | UHRR_C3_1nM | 764152 | 47-54 | NextSeq 2000 |
| RNA033948 | targeted | UHRR_C4_1nM | 672665 | 47-54 | NextSeq 2000 |
| RNA033949 | targeted | UHRR_C4_1nM | 995161 | 47-54 | NextSeq 2000 |
| RNA033964 | targeted | IMR-32_C3_1nM | 865516 | 47-54 | NextSeq 2000 |
| RNA033965 | targeted | IMR-32_C3_1nM | 685457 | 47-54 | NextSeq 2000 |
| RNA033968 | targeted | IMR-32_C4_1nM | 708204 | 47-54 | NextSeq 2000 |
| RNA033969 | targeted | IMR-32_C4_1nM | 1023544 | 47-54 | NextSeq 2000 |

**Supplementary Table 6.** List of 40 PeakPrime designed primers for 40 ERCC spikes.

| Spike Id | Sequence |
| --- | --- |
| ERCC_00003 | TGGGAGGGACTATTCACAGT |
| ERCC_00004 | GCTTGCCTGTTTTTGCCACT |
| ERCC_00012 | AAGTCGGGAAGGAAGCATGG |
| ERCC_00017 | GCTTTTGGCAATACCCAGGC |
| ERCC_00022 | CGATCGCGGAGTTAAGGTCA |
| ERCC_00034 | CGATAGTGGTTGGCTCCTCC |
| ERCC_00035 | CTGAACGAGGGAGTGCTTGT |
| ERCC_00039 | ATGTAAGCTGAATGGGGCCC |
| ERCC_00040 | ATTCTATGGGCAGCGATGGG |
| ERCC_00042 | GGGTCGATGGATGGGTTTGT |
| ERCC_00044 | CGCAATCTATGTTCGCATGC |
| ERCC_00053 | AATCCTTTCCCTGCCTGAGC |
| ERCC_00057 | GGCGAACGTCGTCTGCTATA |
| ERCC_00058 | TAAAGAGGCTCGGCACCATG |
| ERCC_00059 | GCATCGGGTTCACGGCTATA |
| ERCC_00060 | CTAACGATTGGCTCCTCCCC |
| ERCC_00061 | ACGAAGCATGTTGACCGTCT |
| ERCC_00067 | TAATTGTCCGTGCGACGACA |
| ERCC_00069 | TCCGGTTCCAAGCATGACTC |
| ERCC_00071 | CCCGGAGATGAACTACCACG |
| ERCC_00074 | CCTGGAAAAACCTTTGCTGG |
| ERCC_00075 | GGCCCTGGAACAACGGTTAT |
| ERCC_00078 | CTTACACGCTTGCTTGCTCC |
| ERCC_00081 | AGTTGCACGCTGGTACATCA |
| ERCC_00083 | ACCCCCATTGCATAAGCTCC |
| ERCC_00096 | AGCTTCCTGAGGGAGGTCAA |
| ERCC_00097 | TCCAACAGTTTCAGCCAACA |
| ERCC_00104 | CCAACAGAGCTTAAAGCCGT |
| ERCC_00113 | TTTTGGGTTTAGGGGCCTGC |
| ERCC_00116 | TCATGCGACAGTCTCGACAG |
| ERCC_00117 | CCAAAGAACATTGCCCGACG |
| ERCC_00120 | ATCCTCGTCGACCCAACAAC |
| ERCC_00130 | CGACGCTGCAGCCTTTAAAG |
| ERCC_00136 | GGACTCGGACTTCGCAAAGA |
| ERCC_00145 | AGACGCAGGCACAACTCTTT |
| ERCC_00148 | ACCCCTCCAAACGCATTCTT |
| ERCC_00160 | CCTATAGCGAGGCCATTGCA |
| ERCC_00162 | ATTGCAGCCAATCCTGGTGG |
| ERCC_00164 | TAACTAACGGAGCCCCCTCT |
| ERCC_00171 | ACCATTGGATGAAGCCCAGG |
