# Supplementary figures and images for: PeakPrime: a peak-guided primer design pipeline for target enrichment in 3′-end RNA-seq
Source: Bioinform Adv. 2026 Mar 19;6(1):vbag080. doi: 10.1093/bioadv/vbag080 (PMC13034549; doi:10.1093/bioadv/vbag080)

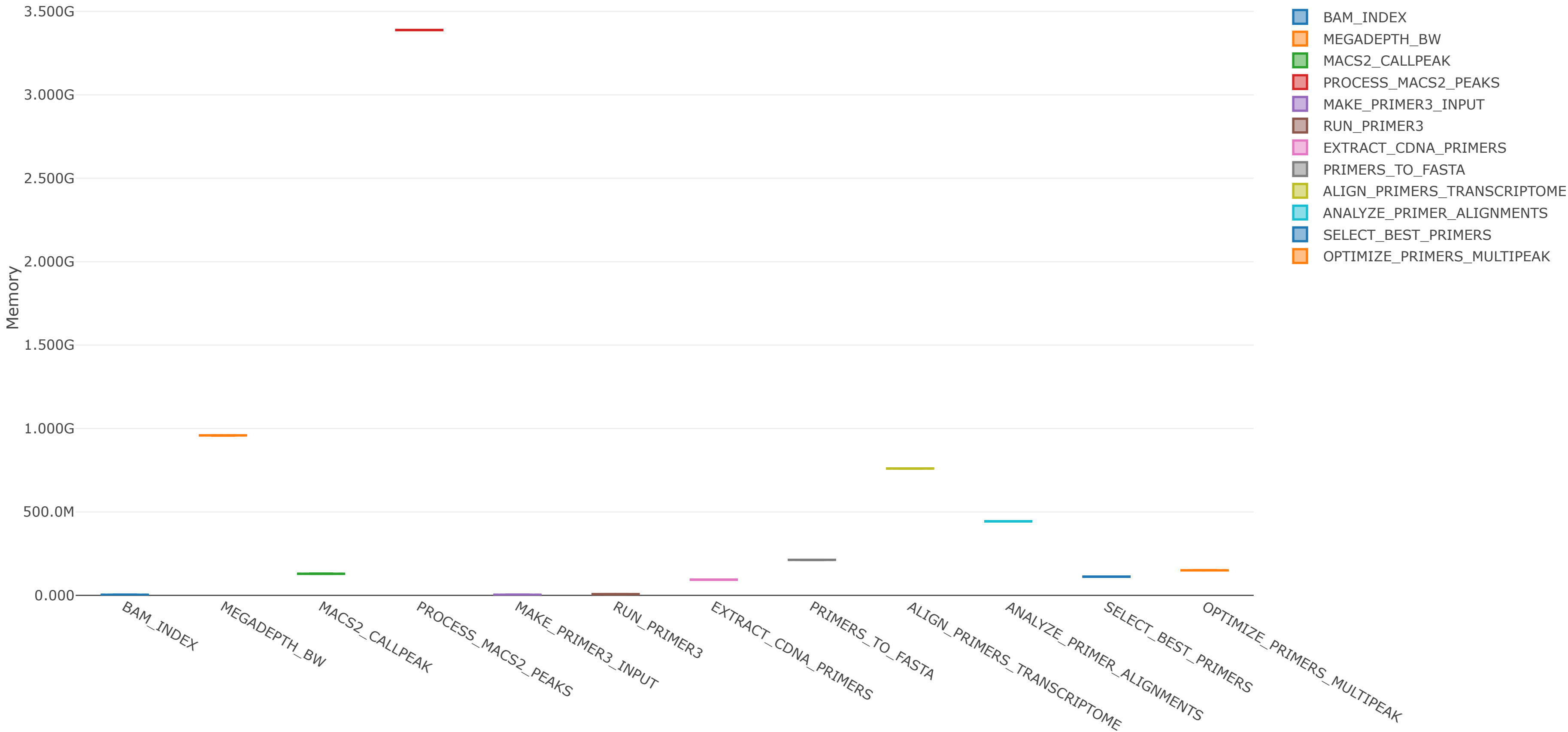

Supplement: vbag080_Supplementary_Data [file vbag080_supplementary_data.zip › SupFig1.pdf]

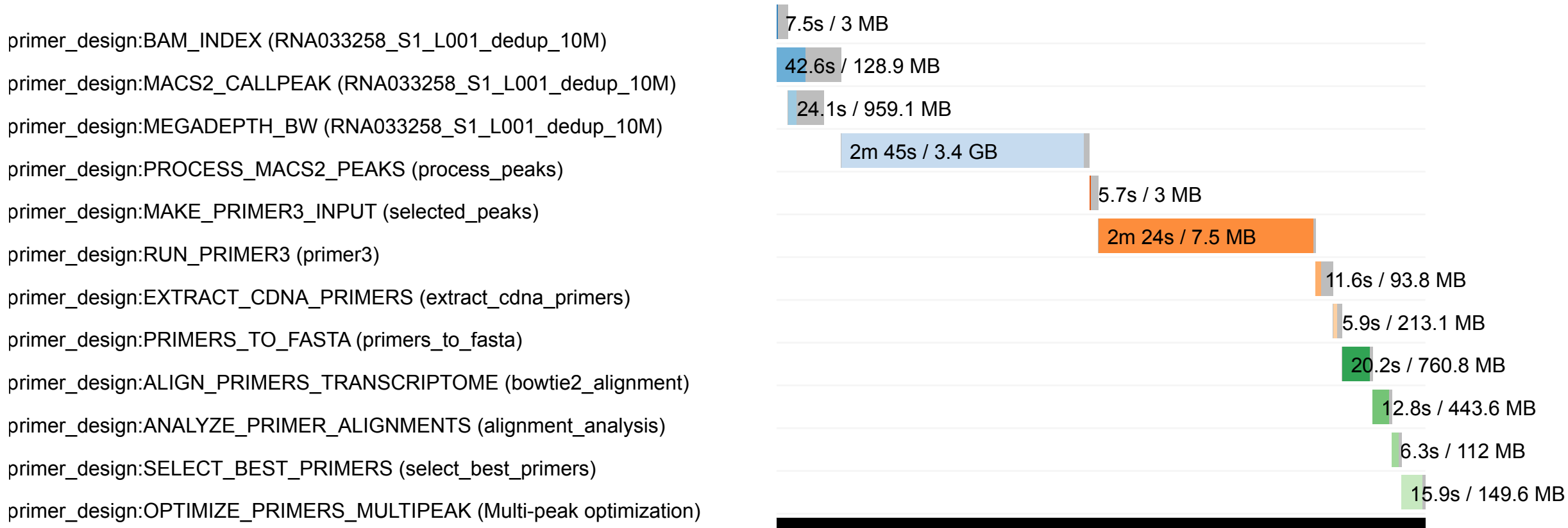

Supplement: vbag080_Supplementary_Data [file vbag080_supplementary_data.zip › SupFig2.pdf]

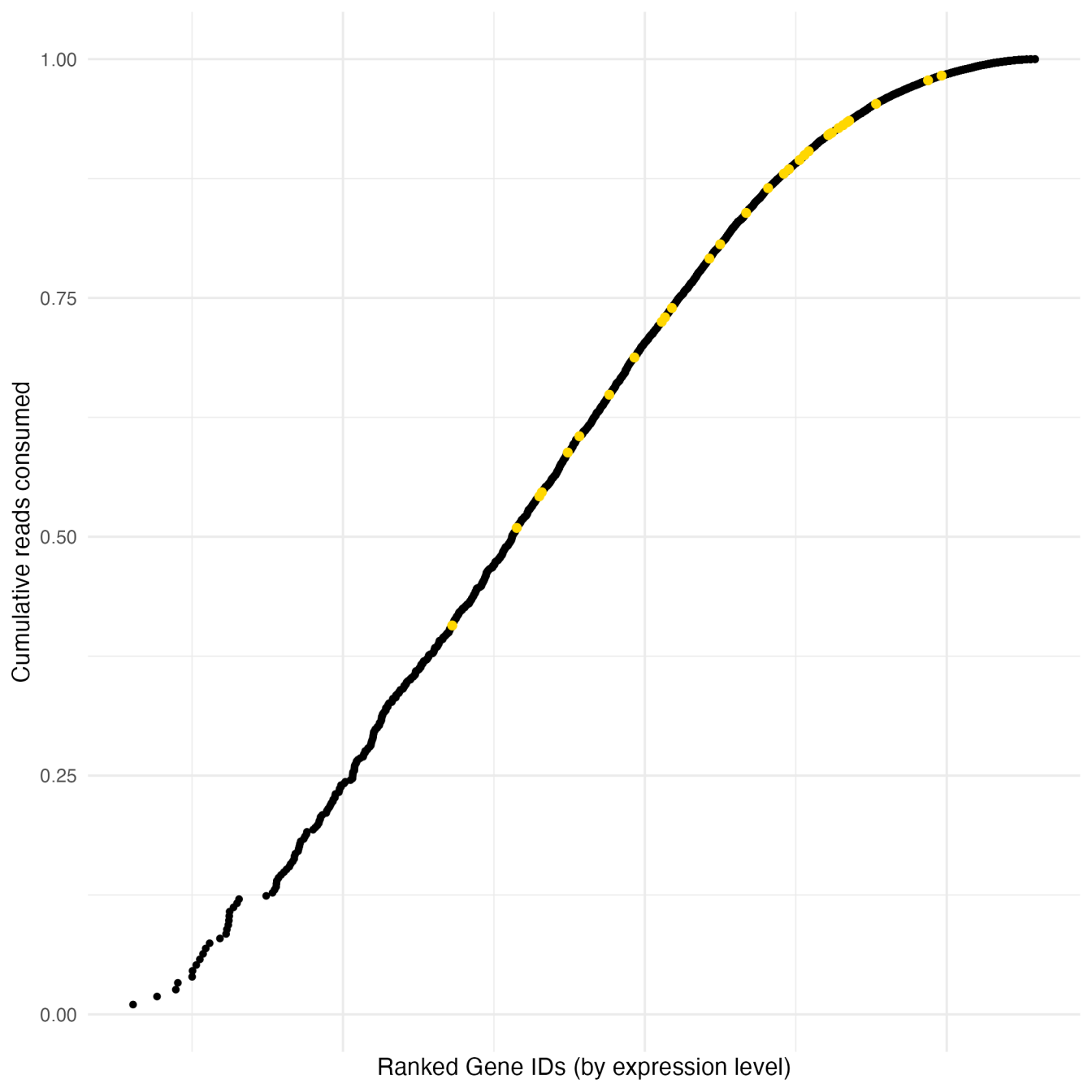

Supplement: vbag080_Supplementary_Data [file vbag080_supplementary_data.zip › SupFig3.pdf]
